# *Enterococcus faecalis* Glycolipids Modulate Lipoprotein-Content of the Bacterial Cell Membrane and Host Immune Response

**DOI:** 10.1371/journal.pone.0132949

**Published:** 2015-07-14

**Authors:** Christian Theilacker, Ann-Kristin Diederich, Andreas Otto, Irina G. Sava, Dominique Wobser, Yinyin Bao, Katrin Hese, Melanie Broszat, Philipp Henneke, Dörte Becher, Johannes Huebner

**Affiliations:** 1 Center for Chronic Immunodeficiency (CCI), University Medical Center Freiburg, Freiburg, Germany; 2 Center for Infectious Disease and Travel Medicine, University Medical Center Freiburg, Freiburg, Germany; 3 Division of Pediatric Infectious Diseases, Dr. von Hauner Children's Hospital, Ludwig-Maximilians-University, Munich, Germany; 4 Institute for Microbiology, Department of Microbial Physiology, Ernst-Moritz-Arndt-University, Greifswald, Germany; 5 Center for Paediatrics and Adolescent Medicine, University Medical Center Freiburg, Freiburg, Germany; 6 Department of Microbiology, Faculty of Biology, Albert-Ludwigs-University, Freiburg, Germany; 7 Research Center for Nutrition and Food Science, Technical University Munich, Freising, Germany; University of Kansas, UNITED STATES

## Abstract

In this study, we investigated the impact of the cell membrane composition of *E*. *faecalis* on its recognition by the host immune system. To this end, we employed an *E*. *faecalis* deletion mutant (Δ*bgsA*) that does not synthesize the major cell membrane glycolipid diglycosyl-diacylglycerol (DGlcDAG). Proteomic analysis revealed that 13 of a total of 21 upregulated surface-associated proteins of *E*. *faecalis* Δ*bgsA* were lipoproteins. This led to a total lipoprotein content in the cell membrane of 35.8% in Δ*bgsA* compared to only 9.4% in wild-type bacteria. Increased lipoprotein content strongly affected the recognition of Δ*bgsA* by mouse macrophages in vitro with an increased stimulation of TNF-α production by heat-fixed bacteria and secreted antigens. Inactivation of the prolipoprotein diacylglycerol transferase (*lgt*) in Δ*bgsA* abrogated TNF-α induction by a Δ*bgsA_lgt* double mutant indicating that lipoproteins mediate increased activation of mouse macrophages by Δ*bgsA*. Heat-fixed Δ*bgsA* bacteria, culture supernatant, or cell membrane lipid extract activated transfected HEK cells in a TLR2-dependent fashion; the same was not true of wild-type bacteria. In mice infected intraperitoneally with a sublethal dose of *E*. *faecalis* we observed a 70% greater mortality in mice infected with Δ*bgsA* compared with wild-type-infected mice. Increased mortality due to Δ*bgsA* infection was associated with elevated plasma levels of the inflammatory cytokines TNF-α, IL-6 and MIP-2. In summary, our results provide evidence that an *E*. *faecalis* mutant lacking its major bilayer forming glycolipid DGlcDAG upregulates lipoprotein expression leading to increased activation of the host innate immune system and virulence in vivo.

## Introduction

In invasive bacterial infections, host inflammation may vary from low-grade to a strong systemic response associated with multi-organ failure and severe sepsis. The differences in the host response are thought to result mainly from activation of the innate immune system by pathogen- and danger-associated molecular patterns. In Gram-positive sepsis, a variety of microbial compounds such as peptidoglycan and its derivatives, bacterial DNA, lipoteichoic acid, and lipoproteins are believed to activate the host immune system [1]. Numerous studies in mice have underlined the role of Toll-like receptor 2 (TLR2) as a major sensor of Gram-positive bacteria, yet its role in vivo is strongly dependent on the specific infectious microorganism [2–6]. In contrast, no clear association has been established between TLR2 variants and susceptibility to Gram-positive infection in humans [7,8].

Several TLR2 ligands have been identified in Gram-positive bacteria, including peptidoglycan, LTA, and lipoproteins/lipopeptides [9]. Studies with mutants of the lipoprotein-acyl transferase (*lgt*) gene and considerations regarding the structure-function relationship suggest that lipopeptides/lipoproteins are the predominant agonists of the TLR2/TLR6 dimer [10,11]. Lipoproteins/lipopeptides are important amphiphiles of the cell membrane in Gram-positive bacteria. They are found both in the cell envelope and culture supernatant [12]. In *Mycobacterium tuberculosis*, membrane-derived vesicles have been described as a vehicle to release lipoproteins into the environment and similar vesicles have also been described for *S*. *aureus* [13,14]. Together with phospholipids, glycolipids, and lipoteichoic acid they constitute the lipid bilayer of the cell membrane.

We have previously studied the impact of cell membrane composition on the virulence of *E*. *faecalis* using mutants deficient in glycolipid biosynthesis. For this purpose we constructed two deletion mutants in *E*. *faecalis* strain 12030 (Δ*bgsA* and Δ*bgsB*) that are defective in the glycosylation of glycolipids [15,16]. Inactivation of *bgsA* leads to a complete loss of DGlcDAG from the cell membrane and accumulation of high concentrations of its precursor molecule monoglycosyl-diacylglycerol (MGlcDAG) [15]. Inactivation of Δ*bgsB*, on the other hand, results in a cell membrane devoid of glycolipids [16]. Both Δ*bgsA* and Δ*bgsB* elaborate a longer poly-glycerophosphate polymer of LTA than wild-type bacteria and show impaired biofilm formation and attachment to colonic epithelial cells. In a mouse bacteremia model, both mutants were cleared more rapidly from the bloodstream [15,16]. Interestingly, defects in glycolipid biosynthesis in Δ*bgsA* and Δ*bgsB* were not associated with changes in the bacterial cell shape or ultrastructure, in the growth rate, or in sensitivity to osmotic stress. This finding was surprising, since the ratio of the bilayer-forming DGlcDAG and the nonbilayer-prone MGlcDAG was shown to be critical for cell membrane architecture and curvature stress in studies using *Acholeplasma laidlawii* [17,18].

Here we examined the consequences of the altered glycolipid composition in Δ*bgsA* on the cell-surface proteome of the bacteria and studied the impact of these changes on the interaction between bacteria and the host immune system. For the investigation of the virulence of glycolipid-deficient *E*. *faecalis* strains we used a mouse peritonitis model that has been validated in several previous studies [19–22]. Our results show that in the absence of DGlcDAG, lipoprotein expression is upregulated in *E*. *faecalis*, which substantially increases the activation of TLR2 and virulence in vivo.

## Materials and Methods

### Bacterial strains, growth conditions, and medium

The bacterial strains and plasmids used in this study are listed in Table 1. Enterococci were cultured in tryptic soy broth (TSB, Merck), M17 broth (Difco Laboratories), Caso Bouillon (Carl Roth), or TSB plus 1% glucose (TSBG) as indicated. In addition, tryptic soy agar or M17 agar plates were used. *Escherichia coli* DH5α and TOP10 (Invitrogen) were cultivated aerobically in LB-broth. For cell culture stimulation studies, bacteria were grown in chemically defined medium (CDM) prepared from endotoxin-free water [23].

**Table 1:** Bacterial strains used and plasmids used in this study.

strain or plasmid	characterization	reference
**strains**		
*E*. *faecalis* 12030	Clinical isolate, strong biofilm producer	[54]
*E*. *faecalis* 12030Δ*bgsA*	(EF2891) *bsgA* deletion mutant	[15]
*E*. *faecalis* 12030Δ*bgsB*	(EF2890) *bsgB* deletion mutant	[25]
*E*. *faecalis* 12030Δ*lgt*	(EF 1748) *lgt* deletion mutant	this study
*E*. *faecalis* 12030Δ*bgsA_lgt*	double *bgsA*-*lgt* deletion mutant	this study
*E*. *faecalis* 12030Δ*bgsB_lgt*	double *bgsB*-*lgt* deletion mutant	this study
*Escherichia coli* DH5α	Gram-negative cloning host	Invitrogen
*Escherichia coli* TOP10	Gram-negative cloning host	Invitrogen
**plasmids**		
pCASPER	Gram-positive, temperature-sensitive mutagenesis vector	[55]
pCRII-TOPO	Gram-negative cloning vector	Invitrogen
pCASPER/Δ*lgt*	pCASPER carrying a *lgt* deletion	this study

### Construction of deletion mutant Δ*lgt*

A non-polar deletion of a portion of gene *lgt* (*EF1748* in *E*. *faecalis* V583, GenBank ID accession number NP_815451) was created using the method described by Cieslewicz et al., [24] with the following modifications: primers 1 and 2 (Table 2) were used to amplify a 503-bp fragment from the region upstream of gene *lgt*, and also the end part of *EF1747*. Primers 3 and 4 were used to amplify a 546-bp fragment downstream of the *lgt* gene and the beginning of *EF1749*. Primers 2 and 3 contain a 21-bp complementary sequence (underlined in Table 2). Overlap extension PCR was used to create a PCR product lacking a portion of gene *EF1748*. The resulting fragment was cloned into Gram-negative cloning vector pCRII-TOPO (Invitrogen) and cut with the restriction enzyme EcoRI (Invitrogen); the resulting fragment was then inserted into shuttle vector pCASPER containing a temperature-sensitive origin of replication. The resulting plasmid, pCASPER/Δ*lgt*, was transformed into *E*. *faecalis 12030* by electroporation, and integrants were selected at a non-permissive temperature (42°C) on TSA plates with kanamycin (1 mg/ml). A single colony was picked, and insertion of plasmid into the chromosome was confirmed by PCR. The integrant was passaged 10 times in liquid culture without antibiotic at the permissive temperature (30°C), and colonies were replica-plated to screen for loss of kanamycin resistance. The excision of the plasmid either creates a reconstituted wild-type strain or leads to an allelic replacement with the deleted sequence in the chromosome. The deletion mutant created was designated *E*. *faecalis* 12030*Δlgt*, containing a 507-bp (169 amino acids) in-frame deletion. The genotype was confirmed by PCR and automated sequencing.

**Table 2:** Primers used in this study.

	name	sequence (5´-3´)^a^
1	pEF1748delF	CCTTGTTCGAGCCCTTTACTT
2	pEF1748OEF	ACTAGCGCGGCCGCTTGCTCCGTTCGTGGCAGCAATTGTTAT
3	pEF1748delR	ACGTCATGAACCTGTTTGGAG
4	pEF1748OER	GGAGCAAGCGGCCGCGCTAGTTAATCTTGCCATTGAAAAGCG

^a^Linkers are underlined.

### Construction of a Δ*bgsA_lgt and* Δ*bgsB_lgt* double mutant

For construction of the double mutants *bgsA_lgt* and *bgsB_lgt* the plasmid pCASPER/Δ*lgt* was transformed into prepared electroporation-competent cells of *E*. *faecalis* 12030Δ*bgsA* or Δ*bgsB* following the procedure described above for the construction of the single Δ*lgt* deletion mutant.

### Preparation of *E*. *faecalis* antigens for stimulation experiments

*E*. *faecalis* strains were grown for 16 h in CDM to stationary phase, collected by centrifugation and washed twice in phosphate buffered saline (PBS). The multiplicity of infection (MOI) for the cell culture experiments was calculated by quantification of colony-forming units (CFU) of serially diluted live bacteria of the respective strain on agar plates, with subsequent adjustment of the suspension of heat-fixed cells to the desired MOI. Cell culture supernatant was filter-sterilized with a 0.2-μm membrane, dialyzed for 24 h against endotoxin-free water, and lyophilized. Bacteria were fixed at 65°C for 60 min. Extraction according to the method of Bligh and Dyer was used to obtain total cell membrane lipids as described previously [15,25]. The concentration of the supernatant and total lipid extracts in the stimulation experiments is expressed as weight per volume.

Lipoproteins were enriched from cell membrane fractions by phase-partitioning with Triton X-114 (TX114) [11,26,27]. To this end, bacterial cells were grown in CDM medium as described above and quantified by serial dilutions and viable counts after culture on agar plates. Bacterial cells were disrupted by vibration with glass beads as described previously [15]. Cell debris and glass beads were pelleted by centrifugation and the supernatant was diluted in TN-buffer (20 mM Tris, 100 mM NaCl, pH 8.0) and passed through a 0.2 μm membrane. For separation of the cell membrane fraction, the filtered supernatant was ultracentrifuged at 50,000 rpm at 4°C for 1 h. The supernatant was discarded and the cell membrane fraction redissolved in TN-buffer plus 2% TX114 (v/v) and incubated at 4°C for 2 h, followed by a second incubation step at 37°C for 30 min. The solution was centrifuged (6,000 rpm, 10 min, 37°C) for phase separation. The lower detergent-soluble phase was carefully collected. The detergent-soluble phase was further purified by adjusting the TX114 concentration to 2% and repeating the phase partitioning, as described above. For removal of TX114, the detergent phase was mixed with 90% ethanol, incubated at -20°C for 18 h and precipitated proteins were collected by centrifugation. After resuspension in TN-buffer the protein concentration of the lipoprotein extracts was determined photometrically and normalized to bacterial cfu.

### Metabolic labeling, isolation of surface-associated proteins

Surface-associated proteins were isolated by biotin labeling and affinity chromatography. Proteins were quantified with the spike-in of a stable isotope-labeled standard. For metabolic labeling the method of Becher *et al*. was used with modifications [28]. *E*. *faecalis* 12030 wild-type and Δ*bgsA* were each grown separately in both labeled and unlabeled rich growth media for *E*. *coli*, (*E*.*coli*-OD5, Silantes) supplemented with 2% glucose, vitamins (p-amino benzoic acid, biotin, folic acid, niacinamide, pantothenate, riboflavin, thiamine), and nucleotides. Cultures were grown at 37°C for 18 h while shaking at 140 rpm. As an internal standard, equal volumes of wild-type and Δ*bgsA* grown to the same OD_600nm_ in ^15^N-labeled medium were mixed. Subsequently, equivalent OD units of wild-type and Δ*bgsA* cell culture grown in unlabeled medium were each mixed with the internal standard and centrifuged.

Cell pellets of wild-type and Δ*bgsA* were each washed once with PBS (pH 8) plus PMSF (1 mM). Cells (0.15 g) were resuspended in 1 ml PBS/PMSF, and Sulfo-N-hydroxysulfosuccinimide-disulfide-Biotin (Sulfo-NHS-SS-Biotin; Thermo Scientific) was added to produce a final concentration of 1 mM. The cell suspension was shaken carefully on ice for 1 h. After centrifugation, biotinylation was stopped by washing cells three times in 1 ml PBS plus 500 mM glycine. Next, cells were resuspended in 1 ml PBS and disrupted with glass beads (0.1 mm diameter) in two cycles at 6,800 rpm for 30 s using a ribolyser (Roche). To obtain cell membrane proteins, cell debris was washed six times with PBS and centrifuged at 45,000 rpm for 1 h at 4°C. Cell debris was resuspended in 0.4 ml PBS/I2-Iodoacetamide (5%) and homogenized with glass beads (0.1 mm diameter) using a ribolyser (6,800 rpm; 2x 30 s). To each sample 100 μl PBS plus 20% CHAPS and 20% Amidosulfobetaine-14 was added, homogenized in a ribolyser (6,800 rpm; 2 x 30 s) and shaken gently for 1 h at 20°C. Cell debris was removed again by centrifugation (14,000 rpm; 15 min; 4°C). Biotinylated proteins were recovered by incubation of the protein lysate with NeutrAvidin-agarose-beads equilibrated in PBS/Nonidet P40 (1%) for 1 h on ice while shaking. Unbound proteins were removed by washing beads four times with PBS/NP-40 plus 6% CHAPS and two times with 1 ml PBS/NP-40 plus 2% SDS. For elution of bound proteins, the disulfide bond of Sulfo-NHS-SS-Biotin was cleaved by incubation with 5% *β*-mercaptoethanol in deionized water for 1 h at 20°C. NeutrAvidin-Agarose-Beads were removed by centrifugation and the supernatant was transferred to 8 ml acetone (-20°C). Elution buffer was once again added to each sample, centrifuged and the resulting supernatant was also added to the acetone-elution-buffer-mix. Proteins were precipitated with acetone overnight at -20°C. Precipitated proteins were centrifuged (8,500 rpm; 30 min; 4°C) and washed with ethanol (98%; 4°C). Finally, the pellet was dried in 6 M urea/2 M thiourea under vacuum (SpeedVac; Bachofer).

### Protein identification and quantification by ESI-LC-MS/MS

Subsequent to preparation of the mixed and labeled protein extracts, samples were subjected to SDS-gel electrophoresis. Resulting gel lanes were cut into equidistant pieces, followed by tryptic in-gel digestion as described elsewhere [29]. The resulting proteolytic digests were subjected to LC-MS/MS analyses as described elsewhere [30]. In brief, peptides were applied to reversed-phase C_18_ chromatography on an EASYnLC system (Thermo Fisher) online coupled to an LTQ-Orbitrap mass spectrometer (Thermo Fisher). For LC-MS analyses a full survey scan in the Orbitrap (m/z 300–2000) with a resolution of 30,000 was followed by MS/MS experiments of the five most abundant precursor ions acquired in the linear trap quadrupole (LTQ) via collision-induced dissociation (CID).

Database searching for light and heavy extracts and subsequent quantitation were done as described in Otto *et al*. [30]. For unambiguous identification data, database searching by Sorcerer- Sequest (version 3.5) relied on an *E*. *faecalis* target-decoy protein sequence database (*E*. *faecalis* V583 (NC_004668.1) including a set of common contaminants). The following parameters were set for database searching: enzyme type, trypsin maximum of two missed trypsin cleavage sites; peptide tolerance, 10 ppm; tolerance for fragment ions, 1amu.; b- and y-ion series; variable modification, oxidation of methionine (15.99 Da). For database searches for ^15^N-labeled peptides, the mass shift of all amino acids completely labeled with^15^N-nitrogen was taken into account. Relative quantification was carried out as described previously [31]. Protein identifications were considered significant for the biological system if the protein was identified in at least two out of three samples in wild type or the mutant. The crude search results served as the base for the further analysis using Census software to obtain quantitative data of ^14^N peaks (sample) and ^15^N peaks (standard) [32].

### Relative quantification of surface-associated proteins by spectral counting

For estimation of relative proportions of proteins within the surface proteome of *E*. *faecalis*, the normalized spectral abundance factor (NSAF) for the light mass traces (^14^N) were calculated as described elsewhere [33]. For calculation of NSAF, spectra from raw data were extracted and searched as described above taking into account only light masses. Scaffold (version Scaffold_4.3.2, Proteome Software Inc.) was then used to validate MS/MS based peptide and protein identifications. Peptide identifications were only accepted if they exceeded specific database search engine thresholds. Sequest identifications required at least deltaCn scores of greater than 0.10 and XCorr scores of greater than 2.2, 3.3, and 3.8 for doubly, triply, and quadruply charged peptides, respectively. Protein identifications were accepted if they contained at least two identified peptides. Proteins that contained similar peptides and could not be differentiated based on MS/MS analysis alone were grouped to satisfy the principles of parsimony. Calculation of NSAF was carried out as described elsewhere [34].

### RAW 264.7 mouse macrophage stimulation

RAW 264.7 macrophages were seeded at a density of 1 x 10^5^ cells/ml in 24-well dishes in endotoxin-free DMEM containing 10% fetal calf serum. Cultures were stimulated with either heat-killed bacteria, lyophilized supernatant or lipoprotein TX114-extracts from bacterial culture for 16 h at 37°C in a 5% humidified CO_2_ environment. After the incubation period, cell culture supernatant was collected and TNF-α production measured by commercial ELISA (R&D Systems) according to the manufacturer’s protocols. A commercial LPS preparation from *E*. *coli* 0111:B4 (Sigma Chemicals) was used as positive control.

### Reporter gene analysis

HEK293 cells (Sigma Aldrich) and the stable cell line HEK-TLR2YFP were used in reporter gene studies as described [2]. In brief, cells were seeded into 96-well tissue-culture plates at a density of 5 x 10^5^ cells/well. Cells were transiently transfected 16 hours later with an ELAM.luc reporter construct with TransIT-LT1 transfection reagent (Mirus Bio). Plasmid pcDNA was used to assure equal amounts of transfected DNA. The following day cells were incubated for 6 h with bacterial cells or cell wall extracts as indicated. After incubation, cultured cells were lysed in passive lysis buffer (Promega) and reporter gene activity was measured using a plate reader luminometer (MicroLumat Plus; Berthold).

### Mouse peritonitis model

The virulence of *E*. *faecalis* strains was evaluated in a mouse peritonitis model. The mice were housed in groups of 5 per cage. All procedures were carried out between 8 a.m. and 18 p.m. in the animals home cage. Fourteen female BALB/C mice (Charles River Laboratories) 6–8 weeks old per group were assigned randomly to infection by intra-peritoneal injection of *E*. *faecalis* strains as indicated. The inoculum for the infection model was grown in TSB for 16 h to stationary phase, washed in PBS, adjusted to the desired concentration and injected intraperitoneally (i.p.) in 200 μl of PBS. The bacterial inoculum was confirmed by plating serial dilutions on agar plates. Mice were monitored twice daily for mortality or signs of illness. If mice had reached an unconscious or moribund state they were euthanized by carbon dioxide inhalation and counted as dead. A moribund condition was defined as impaired mobility, the inability to reach food and water or to keep an upright position, labored breathing or cyanosis, or a hunched position for more than 48 h. Moribund mice were placed in a chamber and CO_2_ was introduced at a displacement rate approximately 20% of the chamber volume per min. The CO_2_ flow was maintained for at least 1 minute after respiratory arrest of the animal. For quantification of bacterial counts and measurement of cytokines, mice (six per group) were sacrificed as described above. Peritoneal lavage fluid (PLF) was obtained by injecting 2 ml of sterile PBS with a 18-gauge needle. Blood was drawn under sterile precautions by cardiac puncture and transferred to heparin tubes. Next, the abdomen was opened, and the right kidney was harvested. All samples were directly placed on ice and processed immediately. The number of *E*. *faecalis* CFU in the PLF, blood, and kidney homogenate was determined. Kidneys were weighed and homogenized at 4°C in 2 ml of TSB with a tissue homogenizer. Serial 10-fold dilutions were made of each sample in TSB, 10 μl of each dilution was plated onto TSA plates and CFUs were enumerated after 18 h of incubation. The leukocytes in the PLF were counted as described elsewhere [22]. PLF supernatant and plasma were stored at -20°C until measurement of the cytokines.

### Ethics statement

All animal experiments were performed in compliance with the German animal protection law (TierSchG). The mice were housed and handled in accordance with good animal practice as defined by FELASA and the national animal welfare body GV-SOLAS. The animal welfare committees of the University of Freiburg (Regierungspraesidium Freiburg Az 35/9185.81/G-07/15) approved all animal experiments. The institutional review board of the University of Freiburg approved the study protocol.

### Statistical analysis

Statistical significance for two-way comparisons was determined by an unpaired t-test as indicated. Analysis of variance (ANOVA) for multigroup comparisons was used on log-transformed data, and the Tukey’s multiple-comparison test was used for posthoc analysis for pairwise comparisons. Survival data were compared using the log-rank (Mantel-Cox) test. To identify the up- and downregulated proteins of the mutant compared to the wild-type, the log2 ratio of each protein quantification was calculated, subtracting the median log2 ratio of the replicates of the wild-type from that of the mutant. The relative quantification of proteins by spectral counting was deemed reliable when its ratio was ascertained in two replicates with not less than two peptides in at least one of the replicates [35]. Statistical results were calculated using the Prism 3 software package. Statistical significance for the NSAF values (mutant versus wild-type) was determined by a t-test in the software Scaffold (version Scaffold_4.3.2, Proteome Software Inc., Portland, OR) with a significance threshold alpha = 0.05.

## Results

### Analysis of enterococcal surface-associated proteins by the proteomics approach

To gain insight into the protein composition of the enterococcal cell-surface, wild-type bacteria and Δ*bgsA* surface-exposed proteins were biotinylated and purified by affinity chromatography prior to SDS-PAGE followed by in-gel digestion (IGD) and analysis by LC-MS/MS (GeLC-MS). Before surface proteins were selectively coupled to Sulfo-NHS-SS-Biotin, ^14^N^15^N metabolic protein labeling was performed during cultivation in CDM. Biotin-labeled proteins were purified by affinity-chromatography and eluted by cleavage of the disulfide bond of the Sulfo-NHS-SS-Biotin by reduction.

This approach led to the unambiguous identification of a total of 210 proteins from wild-type bacteria in 2/3 replicates (S1 Table). Determined by the theoretical prediction rules of subcellular localization (reference 53, 36 and the LocateP DataBase was used (http://www.cmbi.ru.nl/locatep-db/cgi-bin/locatepdb.py)) the surface proteome fraction included 23 lipoproteins, 16 membrane proteins, and seven extracellular proteins containing a signal peptide. One hundred and sixty-four of the biotinylated proteins obtained were annotated with a cytoplasmic subcellular localization.

### In Δ*bgsA* the production of lipoproteins is upregulated

**Fig 1:**
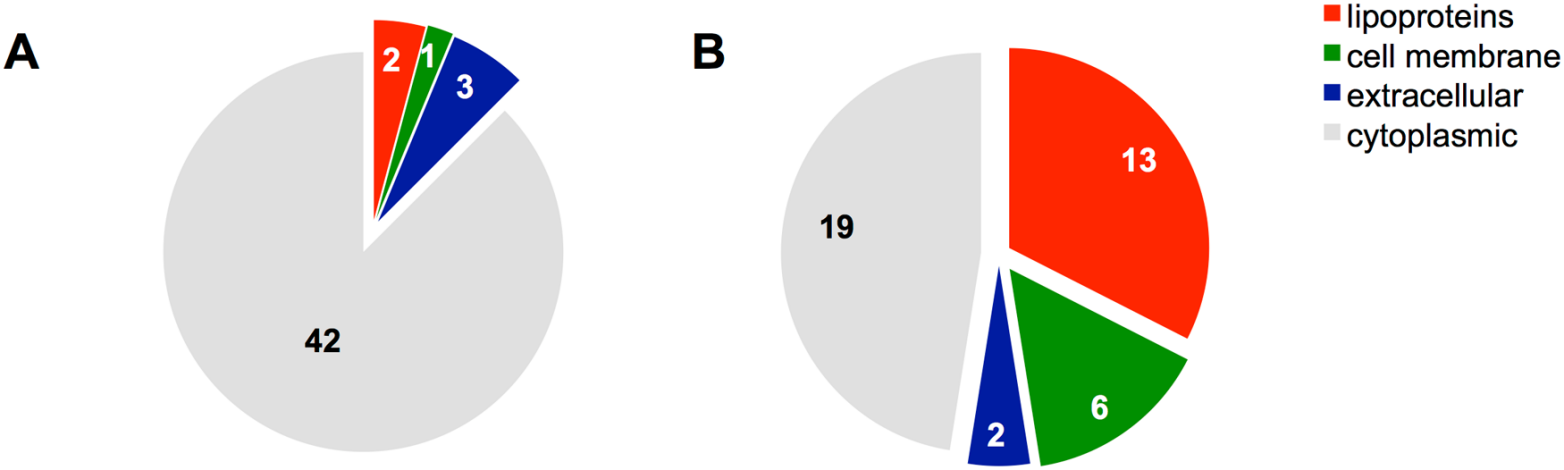
Altered proteins in Δ*bgsA* classified according to subproteomes by bioinformatic identification. Proteins significantly downregulated in Δ*bgsA* compared to *E*. *faecalis* 12030 wild type (A). Upregulated proteins in Δ*bgsA* compared to the wild-type (B). Transmembrane domains were predicted with the TMHMM 2.0 algorithm [53]. Lipoproteins were classified according to Reffuveille *et al*. [36]. For prediction of cell wall, cytoplasmic and extracellular proteins Locate P was used (http://www.cmbi.ru.nl/locatep-db/cgi-bin/locatepdb.py).

The altered composition of cell membrane glycolipids in Δ*bgsA* led to a profound change in the pattern of surface-associated proteins as compared to isogenic wild-type *E*. *faecalis*. A total of 88 proteins were significantly up- or downregulated proteins in Δ*bgsA* according to the statistical testing. Forty showed a significantly increased amount in Δ*bgsA* compared with wild-type bacteria, while 48 proteins were decreased (Table 3). Twenty-one predicted surface-associated proteins were upregulated in Δ*bgsA*. Of those, 13 were predicted to be lipoproteins, six cell membrane proteins and two extracellular proteins (Fig 1). Only six of the downregulated proteins were surface-associated proteins. Strikingly, lipoproteins represented 35.8% of the surface-associated proteins of Δ*bgsA* compared to only 9.4% in wild-type bacteria as quantified by spectral abundance factors (detailed information see S2 Table). Of the five most overexpressed surface-associated proteins, all were lipoproteins and they were upregulated 1.95–12.22-fold in Δ*bgsA* compared to wild type bacteria (Table 3). Altogether, our data suggest that the inactivation of *bgsA* disturbs the equilibrium in the cell membrane that in consequence leads to an increased lipoprotein content.

**Table 3:** Surface-associated proteins present in significantly different amounts in Δ*bgsA* compared to the wild-type. The log2ratio depicts the change of Δ*bgsA* compared to the wild-type.

Protein accession number	*log2 (*Δ*bgsA/wt)*	Annotation	Localization^a^
EF3041	12.222	Pheromone binding protein	lipoprotein
EF1641	3.114	Iron compound ABC transporter, iron compound binding protein	lipoprotein
EF1534	2.870	Peptidyl prolyl cis/trans isomerase	lipoprotein
EF3198	2.258	Lipoprotein. YaeC family	lipoprotein
EF1354	2.230	Pyruvate dehydrogenase complex. E1 component. beta subunit	cytoplasmic
EF1353	2.207	Pyruvate dehydrogenase complex. E1 component. alpha subunit	cytoplasmic
EF2191	1.983	dTDP-4-dehydrorhamnose reductase	cytoplasmic
EF3082	1.955	Iron compound ABC transporter. substrate binding protein	lipoprotein
EF1111	1.842	Signal peptidase I	cell membrane
EF1212	1.828	Transcriptional regulator LytR	cell membrane
EF2156	1.813	Uncharacterized protein	extracellular
EF3062	1.768	Cell shape determining protein MreC	extracellular
EF3120	1.740	Serine threonine protein kinase	cytoplasmic
EF3037	1.602	Glutamyl aminopeptidase	cytoplasmic
EF1677	1.601	Lipoprotein. putative	lipoprotein
EF1340	1.562	Pheromone cAM373 lipoprotein	lipoprotein
EF1191	1.526	DegV family protein	cytoplasmic
EF1759	1.491	Phosphate ABC transporter. phosphate binding protein	lipoprotein
EF3027	1.420	Serine protease DO	cell membrane
EF1523	1.324	Conserved domainl protein	cytoplasmic
EF2656	1.315	Flavoprotein family protein	cytoplasmic
EF0176	1.300	Basic membrane protein family	lipoprotein
EF1416	1.201	Glucose-6-phosphate isomerase	cytoplasmic
EF2697	1.159	Conserved domain protein	cell membrane
EF1753	1.141	Uncharacterized protein	cytoplasmic
EF1045	1.111	6-phosphofructokinase	cytoplasmic
EF0761	1.097	Amino acid ABC transporter. amino acid binding permease protein	cell membrane
EF2496	1.085	Lipoprotein	lipoprotein
EF0685	1.082	Foldase protein PrsA	cell membrane
EF0784	1.079	S-adenosylmethionine synthase	cytoplasmic
EF0949	1.040	Phosphotransacetylase	cytoplasmic
EF2608	0.904	ATP synthase subunit beta	cytoplasmic
EF2610	0.903	ATP synthase subunit alpha	cytoplasmic
EF0863	0.887	Glycine betaine carnitine choline ABC transporter. glycine betaine carnitine choline binding protein	lipoprotein
EF2609	0.794	ATP synthase gamma chain	cytoplasmic
EF1355	0.793	Pyruvate dehydrogenase complex E2 component. dihydrolipoamide acetyltransferase	cytoplasmic
EF3255	0.637	Thiamin biosynthesis lipoprotein ApbE. putative	lipoprotein
EF1961	0.478	Enolase	cytoplasmic
EF2549	0.473	Uracil phosphoribosyltransferase	cytoplasmic
EF2903	0.190	ABC transporter. substrate binding protein	lipoprotein
EF3256	-0.569	Pheromone cAD1 lipoprotein	lipoprotein
EF1548	-0.577	Ribosomal protein S1	cytoplasmic
EF1402	-0.580	Conserved domain protein	cytoplasmic
EF1193	-0.592	DNA binding response regulator VicR	cytoplasmic
EF2932	-0.600	AhpC TSA family protein	cytoplasmic
EF1584	-0.607	Cysteine synthase	cytoplasmic
EF0997	-0.696	Cell division protein FtsZ	cytoplasmic
EF1138	-0.871	Oxidoreductase. aldo-keto reductase family	cytoplasmic
EF2355	-0.934	Chaperone protein ClpB	cytoplasmic
EF1744	-0.956	General stress protein. putative	extracellular
EF1963	-0.992	Phosphoglycerate kinase	cytoplasmic
EF1560	-1.012	Uncharacterized protein	cytoplasmic
EF3054	-1.051	Lipoprotein. putative	lipoprotein
EF0944	-1.059	protein. putative	extracellular
EF0715	-1.107	Trigger factor	cytoplasmic
EF1522	-1.118	RNA polymerase sigma factor RpoD	cytoplasmic
EF1764	-1.171	Ribosomal subunit interface protein	cytoplasmic
EF3230	-1.174	30S ribosomal protein S9	cytoplasmic
EF2607	-1.236	ATP synthase epsilon chain	cytoplasmic
EF2397	-1.314	Elongation factor Ts	cytoplasmic
EF0228	-1.380	Adenylate kinase	cytoplasmic
EF2612	-1.382	ATP synthase subunit b	cytoplasmic
EF2866	-1.384	Probable transcriptional regulatory protein	cytoplasmic
EF0671	-1.412	Xaa-his dipeptidase	cytoplasmic
EF0287	-1.442	Elongation factor P	cytoplasmic
EF2718	-1.468	50S ribosomal protein L1	cytoplasmic
EF0453	-1.605	mC-Ohr family protein	cell membrane
EF0105	-1.613	Ornithine carbamoyltransferase. catabolic	cytoplasmic
EF0770	-1.628	Uncharacterized protein	cytoplasmic
EF0020	-1.639	PTS system. mannose specific IIAB components	cytoplasmic
EF2715	-1.642	50S ribosomal protein L7 L12	cytoplasmic
EF2729	-1.689	Transcription termination antitermination protein nusG	cytoplasmic
EF0709	-1.708	Phosphocarrier protein HPr	cytoplasmic
EF2415	-1.738	Uncharacterized protein	cytoplasmic
EF0220	-1.846	30S ribosomal protein S8	cytoplasmic
EF2719	-1.847	50S ribosomal protein L11	cytoplasmic
EF0079	-1.896	Gls24 protein	cytoplasmic
EF0820	-1.993	50S ribosomal protein L25	cytoplasmic
EF2552	-2.036	Sua5 YciO YrdC YwlC family protein	cytoplasmic
EF2395	-2.093	Ribosome recycling factor	cytoplasmic
EF0394	-2.117	Secreted antigen. putative	extracellular
EF1307	-2.132	Protein GrpE	cytoplasmic
EF0012	-2.132	50S ribosomal protein L9	cytoplasmic
EF0080	-2.160	Gls24 protein	cytoplasmic
EF2925	-2.233	Cold shock domain family protein	cytoplasmic
EF2633	-2.306	60 kDa chaperonin	cytoplasmic
EF1308	-2.341	Chaperone protein DnaK	cytoplasmic
EF0466	-2.345	Glucosamine-6-phosphate deaminase	cytoplasmic

^a^ Transmembrane domains were predicted with the TMHMM 2.0 algorithm [53]. Lipoproteins were classified according to Reffuveille et al. [36]. For prediction of cell wall, cytoplasmatic and extracellular proteins Locate P was used (http://www.cmbi.ru.nl/locatep-db/cgi-bin/locatepdb.py). For detailed data see S1 Table.

### Δ*bgsA* promotes vigorous activation of RAW 264.7 mouse macrophages

**Fig 2:**
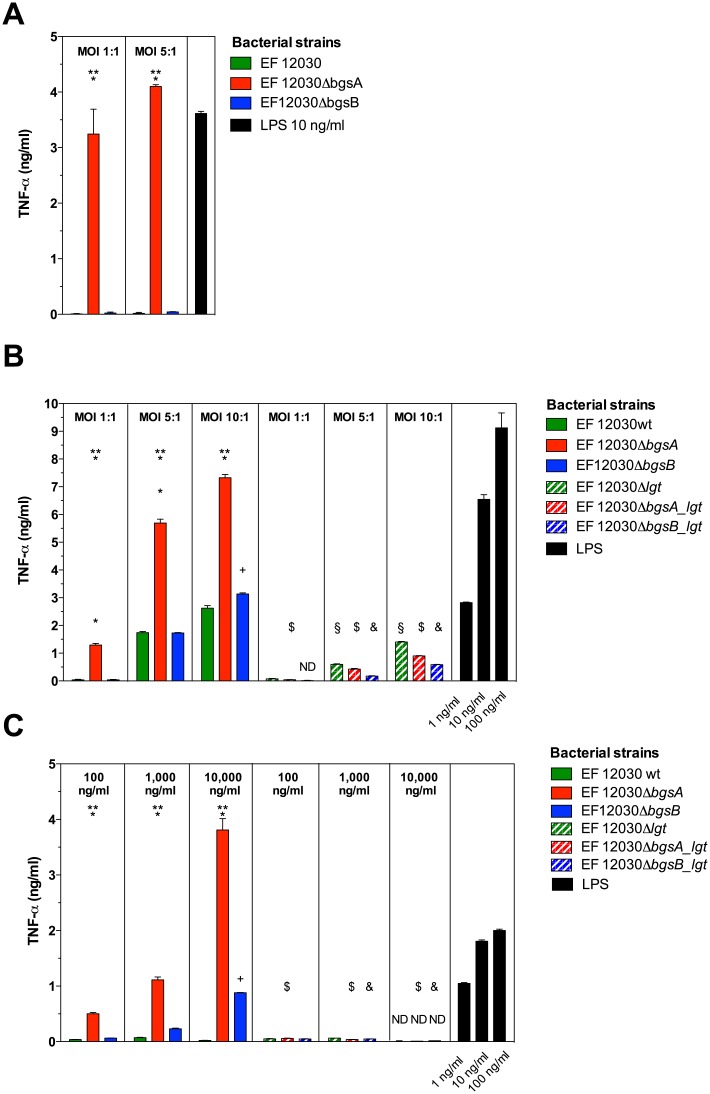
Induction of TNF-α in RAW 264.7 mouse macrophages by *E*. *faecalis* strains. Macrophages were stimulated with live and heat-fixed bacteria at multiplicities of infection (MOI) as indicated (A and B) or with cell-free, dialyzed *E*. *faecalis* supernatants (C). RAW 264.7 macrophages were seeded at a density of 1 x 10^5^ cells/ml in 24-well dishes in endotoxin-free DMEM containing 10% fetal calf serum. Cultures were stimulated at 37°C in a 5% humidified CO_2_ environment for 3 h (A) and 16 h (B), respectively, and supernatant from macrophage culture was analyzed for TNF-α by ELISA. The strains used for stimulation are indicated in the legend. LPS was used as positive control. Data represent mean ± SEM of triplicates. ND = not detected. * p < 0.001 12030 *ΔbgsA* versus 12030 WT, ** p < 0.001 12030 *ΔbgsA* versus 12030 *ΔbgsB*, ^**+**^ p < 0.001 12030 *ΔbgsB* versus 12030 WT. ^§^ p < 0.001 12030 wild type versus 12030 *Δlgt*, ^$^ p < 0.001 12030 *ΔbgsA* versus 12030 *ΔbgsA_lgt*, ^&^ p < 0.001 12030 *ΔbgsB* versus 12030 *ΔbgsB_lgt*. Results were compared by 2-way ANOVA with Bonferroni post-test for pairwise comparisons.

To evaluate the effect of the altered cell-surface composition of Δ*bgsA* on inflammatory responses in vitro, we stimulated RAW 264.7 macrophages in the presence of live and heat-fixed *E*. *faecalis* and measured TNF-α in the cell culture supernatant after 3 h (live bacteria) and 16 h (heat-fixed bacteria and supernatant). In addition to Δ*bgsA* and wild-type bacteria, we used Δ*bgsB* as a second mutant defective in glycolipid biosynthesis in these experiments. At multiplicities of infection between 1:1 and 10:1, Δ*bgsA* induced significantly higher TNF-α concentrations compared to wild-type bacteria and Δ*bgsB*, respectively (Fig 2A and 2B).

*Staphylococcus aureus* lipoproteins are known to be released during growth into the culture medium [12]. We therefore also stimulated RAW 264.7 macrophages with dialyzed culture supernatant of *E*. *faecalis* strains. Supernatant from Δ*bgsA* was a potent inducer of TNF-α production of RAW 264.7 cells in vitro (Fig 2C). While culture supernatant of wild-type bacteria was inactive even at concentrations as high as 10,000 ng/ml (dry weight per volume), Δ*bgsA* stimulated TNF-α production at concentrations a 100-fold lower (Fig 2). Low amounts of TNF-α were also induced by cell culture supernatant from Δ*bgsB* (Fig 2). Together with the proteome analysis, these results demonstrate that lack of DGlcDAG in the cell membrane of Δ*bgsA* not only changes the composition of cell-surface proteome, but also enhances the activation of innate immunity.

### Lipoprotein-enriched cell membrane fractions of Δ*bgsA* but not wild-type cells stimulate TNF-α production of RAW 264.7 macrophages

**Fig 3:**
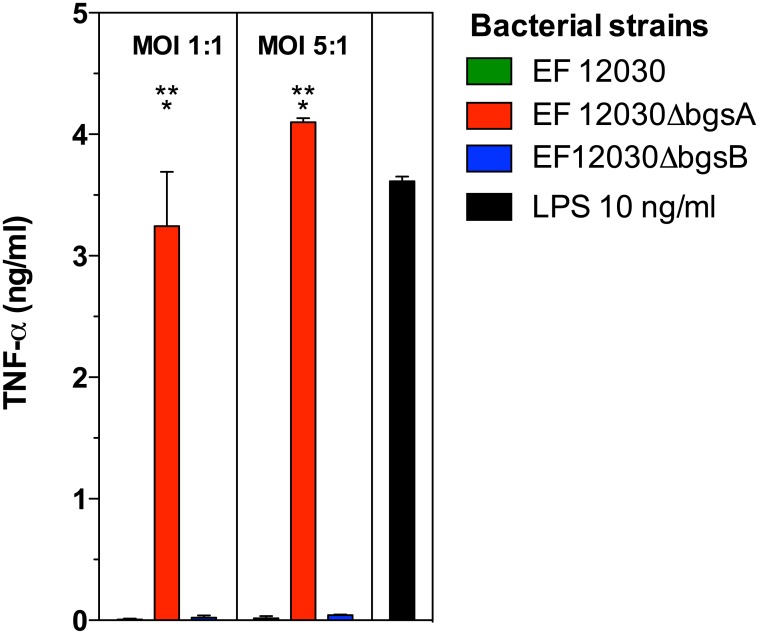
Stimulation of TNF-α production in RAW 264.7 mouse macrophages by lipoprotein-enriched cell membrane fractions of *E*. *faecalis* wild type and *ΔbgsA*. RAW 264.7 cells were incubated with lipoprotein-enriched Triton X-114 extracts from total membrane protein fractions derived from the indicated *E*. *faecalis* strains. The concentration of lipoprotein extracts was measured photometrically and normalized to a bacterial cfu:RAW 264.7 cell ratio of 10,000:1. At 16 h, supernatants were collected and TNF-α concentrations were quantified by ELISA. LPS at a concentration of 100 ng/ml was used as positive control. Data represent mean ± SEM of triplicates. * p < 0.001 12030 *ΔbgsA* versus 12030 WT.

Due to their amphiphilic properties, lipoproteins can be purified from cell membrane fractions by the detergent Triton X-114 [11,26]. MS-shotgun analysis of Triton-extracted total membrane protein fractions from ΔbgsA and wild-type bacteria confirmed a high concentration of lipoproteins of 85% and 63%, respectively, in the extracts (data not shown). The Triton-X114 extracts from ΔbgsA induced an increased TNF-α production compared to the wild-type extracts (Fig 3). Taken together, cell membrane fractions of ΔbgsA highly enriched in lipoprotein are more potent inducers of TNF-α than those from the wild-type strain.

### Inactivation of lipoprotein acylation abrogates induction of TNF-α by wild-type *E*. *faecalis* and *ΔbgsA*

In total, 90 genes that harbor the type II signal sequence typical for lipoproteins have been identified in *E*. *faecalis*, representing about 2.7% of the genome [36]. Similar to other Gram-positive bacteria, inactivation of the prolipoprotein diacylglycerol transferase (*lgt*) in *E*. *faecalis* causes the arrest of lipoprotein maturation at the stage of acylation of the protein, yielding non-acylated lipoproteins [37,38]. In *E*. *faecalis*, the deletion of *lgt* had no effect on bacterial morphology, growth rate, and sensitivity to sodium chloride, different pH condition, or exposure to antibiotics [38]. To corroborate our findings, we constructed a deletion mutant of the *lgt* gene in *E*. *faecalis* 12030 and created the double-deletion mutants EF 12030Δ*bgsA_lgt* and EF 12030Δ*bgsB*_*lgt*. Activation of TNF-α production in macrophages stimulated with *lgt*-mutants or wild-type *E*. *faecalis* was analyzed (Fig 2). Similar to *S*. *aureus* and group B streptococci [2,12,39], impaired protein-lipidation in *E*. *faecalis* 12030 led to a reduction of macrophage activation (Fig 2). Likewise, deletion of *lgt* in Δ*bgsA* and Δ*bgsB* decreased the production of TNF-α by RAW 264.7 macrophages compared to the respective single mutant (Fig 2). Inactivation of the *lgt*-gene in Δ*bgsA* had even stronger effects on TNF-α production in the RAW 264.7 macrophage activation assay if culture supernatant was used as stimulant (Fig 2).

### Δ*bgsA* induces a strong activation of TLR2

**Fig 4:**
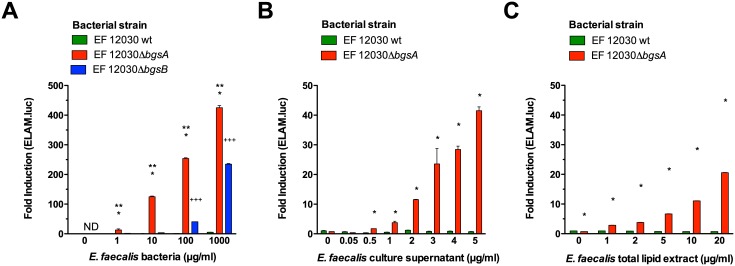
Stimulation of HEK-TLR2 cells transfected with an NF-κB-dependent ELAM-luciferase reporter gene with *E*. *faecalis* antigens. HEK cells were stimulated for 6 h with escalating antigen concentrations as indicated, lysed and luciferase activity was determined by luminometry. The concentration of bacterial cells is expressed as dry weight per ml. Fold-induction denotes stimulated versus non-stimulated luciferase activity. Stimulation with whole bacterial cells (A). Stimulation with dialyzed cell-free culture supernatant (B). Stimulation with *E*. *faecalis* cell membrane total lipid extracts (C). Data are expressed as mean ± SEM of triplicates. ND = not detected. * p < 0.001 12030 *ΔbgsA* versus 12030 WT, ** p < 0.001 12030 *ΔbgsA* versus 12030 *ΔbgsB*, ^**+++**^ p < 0.001 12030 *ΔbgsB* versus 12030 wild type by 2-way ANOVA with Bonferroni post-test for pairwise comparisons.

Next, we wanted to identify the cognate receptor for lipoproteins in Δ*bgsA*. To this end, wild-type bacteria, Δ*bgsA* and Δ*bgsB* were analyzed for NF-κB activation in a NF-κB-dependent luciferase reporter assay in epithelial cells (HEK 293) that stably express the human TLR2 receptor [40]. Whole *E*. *faecalis* wild-type bacteria did not activate NF-κB even at high concentrations (Fig 4). In contrast, Δ*bgsA* induced NF-κB activation at concentrations as low as 1 μg/ml (dry weight). Δ*bgsB* also induced NF-κB, but was a less potent stimulus than Δ*bgsA* (Fig 4). Dialyzed, cell-free bacterial cell culture supernatant of wild-type bacteria also did not induce NF-κB activation, while supernatant from Δ*bgsA* was a strong inducer. Cells were also stimulated with bacterial cell envelope compounds extracted by the Bligh-Dyer method. This method extracts lipids and lipid-containing biomolecules from bacterial or eukaryotic cells [41]. Again, extracts of Δ*bgsA* but not of the wild-type stimulated TLR2 activation (Fig 4). Compared to whole bacterial cells, cell culture supernatant and lipophilic antigens extracts were about 10-fold less potent agonists of the TLR2 receptor (Fig 4). These data establish that *E*. *faecalis* Δ*bgsA* cells and its culture supernatant contain higher levels of TLR2 agonists compared to wild-type bacteria.

### Inactivation of *bgsA* enhances virulence in a mouse bacteremia model

**Fig 5:**
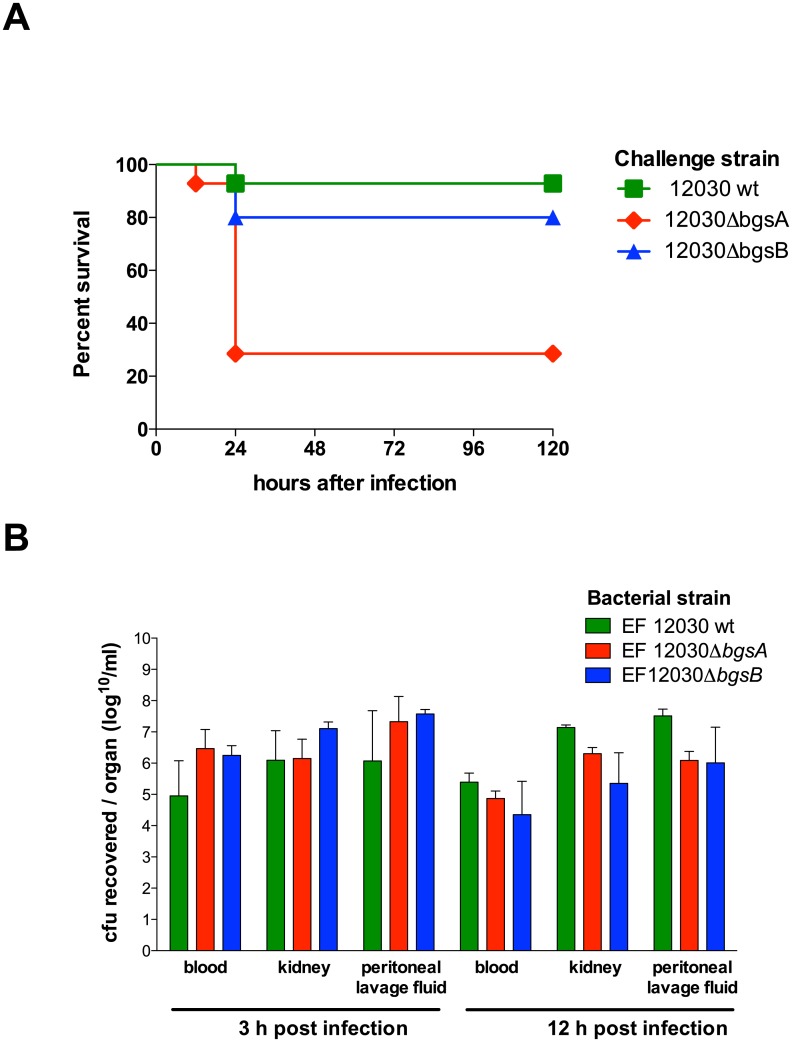
Survival and bacterial load of BALB/c mice after intraperitoneal infection with *E*. *faecalis* strains. Survival after infection with 1.3 x 10^9^ *E*. *faecalis*. P = 0.0096 EF 12030 wild-type vs. Δ*bgsA* determined by the log-rank test (A). Bacterial counts 3 h and 12 h after i.p. challenge (inoculum 2.0 x 10^9^ cfu per mouse, 14 mice per group). The bacterial load is expressed as the log_10_ (cfu) per ml ± SEM (B). P > 0.05 EF 12030 wild-type vs. Δ*bgsA* vs. Δ*bgsB* at 3 h and 12 h by 2-way ANOVA.

TLR-mediated activation of cellular innate immunity during infection is tightly regulated, since over- and underactivation can have detrimental consequences to the host. Given the strong engagement of TLR2 by Δ*bgsA*, we were interested in the effects of the deletion of *bgsA* on enterococcal virulence in a mouse peritonitis model. The LD50 in this model is 5.0 x 10^9^ bacteria, reflecting the relatively low virulence of *E*. *faecalis* in vivo [22]. For the mouse peritonitis model we employed a bacterial dose slightly below the LD50. One of 14 mice died after infection with 1.3 x 10^9^ wild-type bacteria (Fig 5). In contrast, the same inoculum of Δ*bgsA* killed 10 of 14 mice within 24 h (p = 0.0096). There were no subsequent deaths (total observation time five days). No significant difference in mortality between mice infected with Δ*bgsB* and those infected with wild-type *E*. *faecalis* was observed (Fig 5A). We repeated the experiment at a lower inoculum of 3 x 10^8^ bacteria per mouse. With this lower dose, 2 of 8 mice infected with Δ*bgsA* died within the observation period (5 days), while all mice infected with the wild-type survived (p = 0.14).

### Peritonitis caused by EF12030Δ*bgsA* is associated with increased pro-inflammatory cytokines

To better understand excess mortality in peritonitis induced by Δ*bgsA*, we infected mice with an inoculum similar to that used in the survival model (2.0 x 10^9^) and quantified the bacterial concentration in the peritoneal cavity, blood, and kidneys at 3 and 12 hours after infection (Fig 5B). No significant difference in bacterial load was noted at corresponding time points. Thus, Δ*bgsA* does not appear to impede bacterial clearance during peritonitis, and differences in bacterial load cannot explain the differences in mortality.

**Fig 6:**
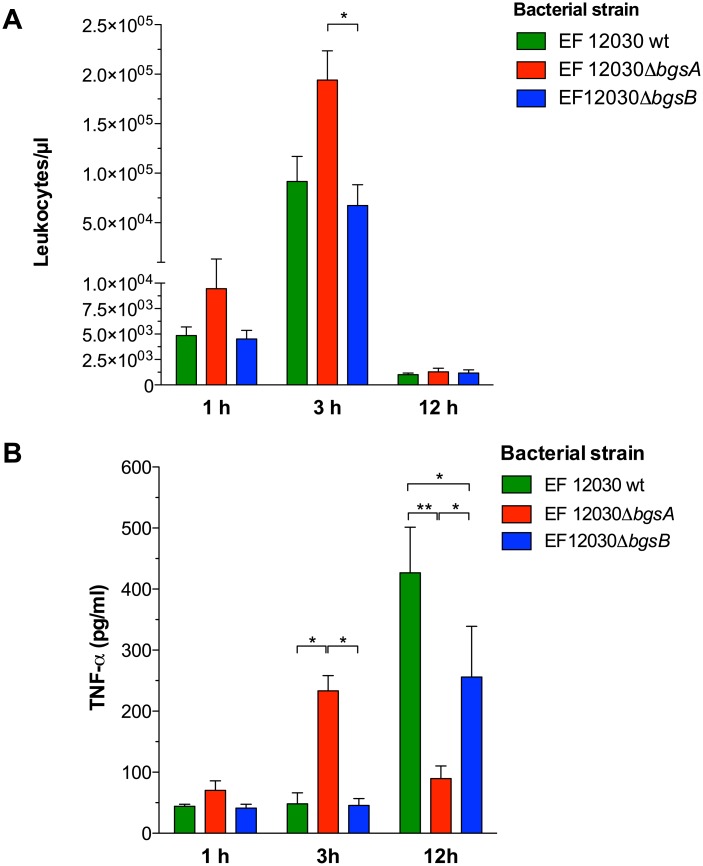
Leukocyte count and TNF-α expression in the PLF 1, 3, and 12 h after intraperitoneal infection with *E*. *faecalis* strains. Number of leukocytes in the PLF (A). TNF-α in the PLF (B). * p < 0.05, ** p < 0.001, by 2-way ANOVA. Data represent means ± SEM of individual mice (8 mice per group).

**Fig 7:**
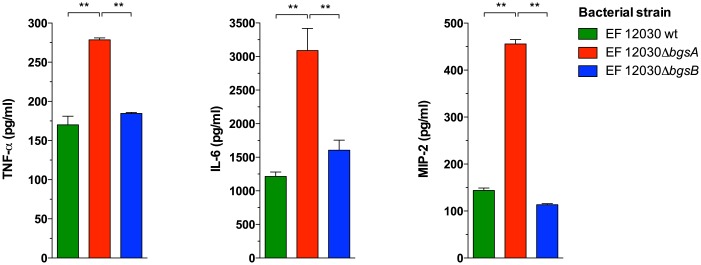
Inflammatory cytokines/chemokines in the plasma 1 h after intraperitoneal infection with *E*. *faecalis* strains. * p < 0.05, ** p < 0.001, by 2-way ANOVA. Data represent means ± SEM of individual cytokine levels of 8 mice.

We also measured leukocytes and inflammatory cytokines during *E*. *faecalis* peritonitis. Three hours after infection, more leukocytes were recruited to the peritoneal cavity in animals infected with Δ*bgsA* than in those infected with wild-type bacteria or Δ*bgsB* (Fig 6). To determine whether a dysregulated inflammatory response was involved in the increased mortality caused by Δ*bgsA*, we measured cytokine concentrations in the peritoneal fluid and blood. One hour after infection with Δ*bgsA*, plasma concentrations of TNF-α, IL-6, and MIP-2 were significantly increased in mice infected with Δ*bgsA* as compared to mice infected with wild-type bacteria or Δ*bgsB* (Fig 7). Infection with Δ*bgsA* resulted also in significantly increased TNF-α concentrations in the peritoneal lavage fluid 3 h after infection compared to mice with peritonitis caused by wild-type bacteria or Δ*bgsB* (Fig 6B). At 12 h after infection, the kinetics of TNF-α production in the peritoneum had reversed: while low amounts of TNF-α were found in mice infected with Δ*bgsA*, mice infected with the wild-type strain or Δ*bgsB* displayed elevated levels of this cytokine (Fig 6B). These results show a correlation between mortality caused by Δ*bgsA* and increased production of inflammatory cytokines at early time points.

## Discussion

Our current work demonstrates the consequences of a disturbed cell membrane glycolipid homeostasis on the expression of lipoproteins in the cell envelope and the activation of the innate immune system by *E*. *faecalis*. Inactivation of the *bgsA* gene in *E*. *faecalis* led to a more than 3-fold higher lipoprotein-content in Δ*bgsA* compared to wild-type bacteria. Δ*bgsA* antigens were more potent activators of mouse macrophages and much stronger agonists of the TLR2 receptor. Genetic inactivation of the biosynthesis of lipoprotein strongly reduced the potency of Δ*bgsA* to stimulate macrophages. Conversely, lipoprotein-enriched Triton X114 extracts from total membrane fractions of Δ*bgsA* were stronger activators of mouse macrophages than wild type extracts. The alteration of the cell-surface proteome by inactivation of *bgsA* also enhanced the virulence of *E*. *faecalis* in a mouse peritonitis model.

Immune recognition of enterococci during mouse peritonitis has been described in detail by Leendertse and coworkers [20,22]. Peritoneal infection by *Enterococcus faecium* is sensed predominantly by macrophages via TLR2 which then secrete IL6, TNF-α, MIP-2, and KC during early infection [20,22]. A consecutive influx of neutrophils into the peritoneal cavity then leads to the clearance of the bacteria [20,22]. Working with an infection model similar to the one used by Leendertse, we were able to examine the consequences of overstimulation of the TLR2 system on the natural course of peritoneal infection. Peritonitis due to Δ*bgsA* led to an excessive induction of TNF-α, IL6, and MIP-2 accompanied by a higher influx of leukocytes in the peritoneum and to increased mortality of infected mice. Our results confirm previous studies which have shown that the overproduction of chemokines such as MIP-2 contributes to mortality in animal models of polymicrobial sepsis [42–44].

It is interesting that the effects of the inactivation of *bgsA* and a de-repressed lipoprotein production on the virulence of *E*. *faecalis* are highly dependent on the type of infection. We reported previously that inactivation of *bgsA* reduces biofilm formation and adherence to colonic cells, while the mutation improves the ability of *E*. *faecalis* to colonize the urinary tract [15,45]. In a mouse bacteremia model, Δ*bgsA* was cleared more rapidly from the bloodstream than the wild-type strain [15]. Yet during peritoneal infection as reported here, inactivation of *bgsA* induced stronger inflammation and led to higher mortality. Because of the pleiotropic phenotype of Δ*bgsA*, these results have to be interpreted with caution [15]. Nevertheless, repression of lipoprotein production by *bgsA* may be advantageous in some host compartments to escape detection by the host immune system while in others, like the peritoneum, virulence is suppressed. Since colonization of the gastrointestinal tract is the default ecological niche of *E*. *faecalis*, repression of lipoprotein production by *bgsA* probably improves overall fitness by evasion of the mucosal immune system. A similar strategy to subvert recognition by the innate immune system has been described by the intracellular pathogen *Francisella novicida* [46]. The *F*. *novicida* protein FTN_0757 specifically represses the production of its lipoprotein FTN_1103 and deletion of *FTN_0757* leads to stronger stimulation of TLR2 and induction of higher levels of inflammatory cytokines compared to wild-type bacteria [46]. Hence, FTN_0757 was suggested to function as an immune escape mechanism in *F*. *novicida*. Furthermore, in *S*. *aureus*, capsule expression and formation of small colony variants has been described as a means to downregulate TLR2 activation [47]

Our study does not reveal a clear mechanism how the glycolipid mix of the cell membrane leads to an upregulation of lipoprotein concentration. Studies on the role of glycolipids in membrane physiology, however, point to secondary adjustments of the lipid composition in Δ*bgsA* to restore cell membrane homeostasis [48]. The biophysical properties of membrane lipids are determined by the size of their polar head groups in relation to the hydrophobic acyl-glycerol backbone. MGlcDAG with its smaller polar head group forms inverted nonlamellar structures as opposed to the bilayer conformation of DGlcDAG [49]. This results in a larger negative curvature and increased bilayer curvature stress for membranes composed of MGlcDAG as compared to DGlcDAG [49]. Cell membranes of Δ*bgsA* only contain MGlcDAG and the presence of additional, bilayer forming amphiphiles are most likely needed to dilute the concentration of the non-bilayer forming glycolipid. In *Streptococcus pneumoniae*, for example, deletion of glycosyltransferase *cpoA* leads to an arrest of the synthesis of galactosyl-glucosyl-diacylglycerol and to a secondary increase in the proportion of phosphatidylglycerol to cardiolipin [50]. Lipoproteins also contain large polar head groups and could potentially act as bilayer-prone amphiphiles in the cell membrane of Δ*bgsA*. Studies *in Acholeplasma laidlawii* suggest that the activities of the interfacial glycosyltransferases that synthesize MGlcDAG and DGlcDAG are regulated by the physical properties of the membrane containing their substrates [48,51]. Hence, biochemical regulation possibly also governs the compensatory increase of synthesis of lipoproteins in Δ*bgsA*. This model could also explain why inactivation of Δ*bgsB* does not cause enhanced activation of the innate immunity or promotes virulence in peritonitis. Mutation of *bgsB* in *E*. *faecalis* causes a complete arrest of glycolipid synthesis [25] and the mutant therefore does not overproduce toxic non-bilayer forming glycolipids. Hence, fewer adaptive changes to the cell envelope maybe needed to maintain membrane homeostasis.

Another question raised by our study is, if TLR2 activation by Δ*bgsA* is mediated by a global upregulation of lipoproteins or by one or more specific lipoproteins that act as dominant inducers of TLR2. Upregulation clearly affected lipoproteins unevenly in Δ*bgsA*. While a total of 12 lipoproteins were upregulated between 0.19 and 3.11-fold compared to wild-type levels, the expression of EF3041 was increased over 12-fold. Theoretical considerations as well as experimental studies, however, do not support the theory that certain lipoproteins act as dominant inducers of TLR2. Modeling studies of the crystallized structures of the TLR2-TLR6-lipopeptide complex suggest that TLR2 activation by lipopeptides is mediated only by a small and highly conserved structural motif of two ester-bound acyl-chains of at least 12 carbons each and the first two N-terminal amino acids of the polypeptide chain [52]. According to this model, the structure of the polypeptide chain beyond the second amino acid is negligible for the potency of Iipopeptide or lipoprotein to engage TLR2. Furthermore, in vitro studies of the *FTN_0757* deletion mutant in *F*. *novicida* show that equimolar concentrations of lipoproteins from the wild-type strain and Δ*FTN_0757* had a similar potency as TLR2 activators [46].

Taken together, our findings reveal an intimate interplay between the concentration of the bilayer-forming glycolipid DGlcDAG, the expression of lipoproteins, and activation of the host immune system. We show that deletion of *bgsA* leads to upregulation of bacterial lipoproteins and strongly enhances host inflammatory response and virulence in enterococcal peritonitis.

## Supporting Information

S1 Table List of identified proteins by proteomic analysis. The table includes all protein quantification data in “summary”-tab as well as the number of peptides identified in each single analysis in the tabs WT 1/2/3 and *bgsA* 1/2/3. Protein identifications were considered significant for the biological system if the protein was identified in at least two out of three samples in wild type or the mutant.
(XLSX)

S2 Table List of proteins quantified by spectral abundance factors. (XLSX)

## References

[1] Mogensen TH (2009) Pathogen recognition and inflammatory signaling in innate immune defenses. Clin Microbiol Rev 22: 240–273. 10.1128/CMR.00046-08 19366914 PMC2668232

[2] Henneke P, Dramsi S, Mancuso G, Chraibi K, Pellegrini E, Theilacker C, et al (2008) Lipoproteins are critical TLR2 activating toxins in group B streptococcal sepsis. J Immunol 180: 6149–6158. 18424736 10.4049/jimmunol.180.9.6149

[3] Knapp S, Wieland CW, van 't Veer C, Takeuchi O, Akira S, Florquin S, et al (2004) Toll-like receptor 2 plays a role in the early inflammatory response to murine pneumococcal pneumonia but does not contribute to antibacterial defense. J Immunol 172: 3132–3138. 14978119 10.4049/jimmunol.172.5.3132

[4] Yimin, Kohanawa M, Zhao S, Ozaki M, Haga S, Nan G, et al (2013) Contribution of Toll-Like Receptor 2 to the Innate Response against *Staphylococcus aureus* Infection in Mice. PLoS ONE 8: e74287 10.1371/journal.pone.0074287 24058538 PMC3772844

[5] Echchannaoui H, Frei K, Schnell C, Leib SL, Zimmerli W, Landmann R (2002) Toll-like receptor 2-deficient mice are highly susceptible to *Streptococcus pneumoniae* meningitis because of reduced bacterial clearing and enhanced inflammation. J Infect Dis 186: 798–806. 10.1086/342845 12198614

[6] Mancuso G, Midiri A, Beninati C, Biondo C, Galbo R, Akira S, et al (2004) Dual role of TLR2 and myeloid differentiation factor 88 in a mouse model of invasive group B streptococcal disease. J Immunol 172: 6324–6329. 15128822 10.4049/jimmunol.172.10.6324

[7] Brouwer MC, de Gans J, Heckenberg SGB, Zwinderman AH, Van Der Poll T, van de Beek D (2009) Host genetic susceptibility to pneumococcal and meningococcal disease: a systematic review and meta-analysis. Lancet Infect Dis 9: 31–44. 10.1016/S1473-3099(08)70261-5 19036641

[8] Casanova J-L, Abel L, Quintana-Murci L (2011) Human TLRs and IL-1Rs in host defense: natural insights from evolutionary, epidemiological, and clinical genetics. Annu Rev Immunol 29: 447–491. 10.1146/annurev-immunol-030409-101335 21219179

[9] Kawai T, Akira S (2010) The role of pattern-recognition receptors in innate immunity: update on Toll-like receptors. Nat Immunol 11: 373–384. 10.1038/ni.1863 20404851

[10] Zähringer U, Lindner B, Inamura S, Heine H, Alexander C (2008) TLR2—promiscuous or specific? A critical re-evaluation of a receptor expressing apparent broad specificity. Immunobiology 213: 205–224. 10.1016/j.imbio.2008.02.005 18406368

[11] Hashimoto M, Tawaratsumida K, Kariya H, Kiyohara A, Suda Y, Krikae F, et al (2006) Not lipoteichoic acid but lipoproteins appear to be the dominant immunobiologically active compounds in *Staphylococcus aureus* . J Immunol 177: 3162–3169. 16920954 10.4049/jimmunol.177.5.3162

[12] Stoll H, Dengjel J, Nerz C, Götz F (2005) *Staphylococcus aureus* deficient in lipidation of prelipoproteins is attenuated in growth and immune activation. Infect Immun 73: 2411–2423. 10.1128/IAI.73.4.2411-2423.2005 15784587 PMC1087423

[13] Prados-Rosales R, Baena A, Martinez LR, Luque-Garcia J, Kalscheuer R, Veeraraghavan U, et al (2011) Mycobacteria release active membrane vesicles that modulate immune responses in a TLR2-dependent manner in mice. J Clin Invest 121: 1471–1483. 10.1172/JCI44261 21364279 PMC3069770

[14] Gurung M, Moon DC, Choi CW, Lee JH, Bae YC, Kim J, et al (2011) *Staphylococcus aureus* produces membrane-derived vesicles that induce host cell death. PLoS ONE 6: e27958 10.1371/journal.pone.0027958 22114730 PMC3218073

[15] Theilacker C, Sanchez-Carballo P, Toma I, Fabretti F, Sava I, Kropec A, et al (2009) Glycolipids are involved in biofilm accumulation and prolonged bacteraemia in *Enterococcus faecalis* . Mol Microbiol 71: 1055–1069. 10.1111/j.1365-2958.2009.06587.x 19170884

[16] Theilacker C, Sava I, Sanchez-Carballo P, Bao Y, Kropec A, Grohmann E, et al (2011) Deletion of the glycosyltransferase *bgsB* of *Enterococcus faecalis* leads to a complete loss of glycolipids from the cell membrane and to impaired biofilm formation. BMC Microbiol 11: 67 10.1186/1471-2180-11-67 21470413 PMC3083329

[17] Edman M, Berg S, Storm P, Wikström M, Vikström S, Ohman A, et al (2003) Structural features of glycosyltransferases synthesizing major bilayer and nonbilayer-prone membrane lipids in *Acholeplasma laidlawii* and *Streptococcus pneumoniae* . J Biol Chem 278: 8420–8428. 10.1074/jbc.M211492200 12464611

[18] Wikström M, Xie J, Bogdanov M, Mileykovskaya E, Heacock P, Wislander A, et al (2004) Monoglucosyldiacylglycerol, a foreign lipid, can substitute for phosphatidylethanolamine in essential membrane-associated functions in *Escherichia coli* . J Biol Chem 279: 10484–10493. 10.1074/jbc.M310183200 14688287

[19] Leendertse M, Willems RJL, Giebelen IAJ, Roelofs JJTH, Bonten MJM, van der Poll T (2009) Neutrophils are essential for rapid clearance of *Enterococcus faecium* in mice. Infect Immun 77: 485–491. 10.1128/IAI.00863-08 19001080 PMC2612258

[20] Leendertse M, Willems RJL, Giebelen IAJ, Roelofs JJTH, Van Rooijen N, Bonten MJM, et al (2009) Peritoneal macrophages are important for the early containment of Enterococcus faecium peritonitis in mice. Innate Immunity 15: 3–12. 10.1177/1753425908100238 19201820

[21] Leendertse M, Willems RJL, Flierman R, de Vos AF, Bonten MJM, van der Poll T (2010) The complement system facilitates clearance of *Enterococcus faecium* during murine peritonitis. J Infect Dis 201: 544–552. 10.1086/650341 20064073

[22] Leendertse M, Willems RJL, Giebelen IAJ, van den Pangaart PS, Wiersinga WJ, de Vos AF, et al (2008) TLR2-dependent MyD88 signaling contributes to early host defense in murine *Enterococcus faecium* peritonitis. J Immunol 180: 4865–4874. 18354210 10.4049/jimmunol.180.7.4865

[23] Paoletti LC, Ross RA, Johnson KD (1996) Cell growth rate regulates expression of group B *Streptococcus* type III capsular polysaccharide. Infect Immun 64: 1220–1226. 8606082 10.1128/iai.64.4.1220-1226.1996 PMC173907

[24] Cieslewicz MJ, Kasper DL, Wang Y, Wessels MR (2001) Functional analysis in type Ia group B *Streptococcus* of a cluster of genes involved in extracellular polysaccharide production by diverse species of streptococci. J Biol Chem 276: 139–146. 10.1074/jbc.M005702200 11027683

[25] Bychowska A, Theilacker C, Czerwicka M, Marszewska K, Huebner J, Holst O, et al (2011) Chemical structure of wall teichoic acid isolated from *Enterococcus faecium* strain U0317. Carbohydr Res 346: 2816–2819. 10.1016/j.carres.2011.09.026 22024569

[26] Brusca JS, Radolf JD (1994) Isolation of integral membrane proteins by phase partitioning with Triton X-114. Meth Enzymol 228: 182–193. 8047007 10.1016/0076-6879(94)28019-3

[27] Hashimoto M, Tawaratsumida K, Kariya H, Aoyama K, Tamura T, Suda Y (2006) Lipoprotein is a predominant Toll-like receptor 2 ligand in *Staphylococcus aureus* cell wall components. Int Immunol 18: 355–362. 10.1093/intimm/dxh374 16373361

[28] Becher D, Hempel K, Sievers S, Zühlke D, Pané-Farré J, Otto A, et al (2009) A proteomic view of an important human pathogen—towards the quantification of the entire *Staphylococcus aureus* proteome. PLoS ONE 4: e8176 10.1371/journal.pone.0008176 19997597 PMC2781549

[29] Dreisbach A, Otto A, Becher D, Hammer E, Teumer A, Gouw JW, et al (2008) Monitoring of changes in the membrane proteome during stationary phase adaptation of *Bacillus subtilis* using in vivo labeling techniques. Proteomics 8: 2062–2076. 10.1002/pmic.200701081 18491319

[30] Otto A, Bernhardt J, Meyer H, Schaffer M, Herbst F-A, Siebourg J, et al (2010) Systems-wide temporal proteomic profiling in glucose-starved *Bacillus subtilis* . Nat Commun 1: 137 10.1038/ncomms1137 21266987 PMC3105300

[31] MacCoss MJ, Wu CC, Liu H, Sadygov R, Yates JR (2003) A correlation algorithm for the automated quantitative analysis of shotgun proteomics data. Analytical chemistry 75: 6912–6921. 10.1021/ac034790h 14670053

[32] Park SK, Venable JD, Xu T, Yates JR (2008) A quantitative analysis software tool for mass spectrometry-based proteomics. Nat Methods 5: 319–322. 10.1038/nmeth.1195 18345006 PMC3509211

[33] Zybailov B, Mosley AL, Sardiu ME, Coleman MK, Florens L, Washburn MP (2006) Statistical analysis of membrane proteome expression changes in *Saccharomyces cerevisiae* . J Proteome Res 5: 2339–2347. 10.1021/pr060161n 16944946

[34] Zhang Y, Wen Z, Washburn MP, Florens L (2010) Refinements to label free proteome quantitation: how to deal with peptides shared by multiple proteins. 82: 2272–2281. Anal Chem 82:2272–2281. 10.1021/ac9023999 20166708

[35] Sievers S, Ernst CM, Geiger T, Hecker M, Wolz C, Becher D, et al (2010) Changing the phospholipid composition of *Staphylococcus aureus* causes distinct changes in membrane proteome and membrane-sensory regulators. Proteomics 10: 1685–1693. 10.1002/pmic.200900772 20162562

[36] Reffuveille F, Leneveu C, Chevalier S, Auffray Y, Rincé A (2011) Lipoproteins of *Enterococcus faecalis*: bioinformatic identification, expression analysis and relation to virulence. Microbiology 157: 3001–3013. 10.1099/mic.0.053314-0 21903750

[37] Hutchings MI, Palmer T, Harrington DJ, Sutcliffe IC (2009) Lipoprotein biogenesis in Gram-positive bacteria: knowing when to hold “em, knowing when to fold”em. 17: 13–21. 10.1016/j.tim.2008.10.001 19059780

[38] Reffuveille F, Serror P, Chevalier S, Budin-Verneuil A, Ladjouzi R, Bernay B, et al (2012) The prolipoprotein diacylglyceryl transferase (Lgt) of *Enterococcus faecalis* contributes to virulence. Microbiology 158: 816–825. 10.1099/mic.0.055319-0 22135097

[39] Bubeck Wardenburg J, Williams WA, Missiakas D (2006) Host defenses against *Staphylococcus aureus* infection require recognition of bacterial lipoproteins. Proc Natl Acad Sci USA 103: 13831–13836. 10.1073/pnas.0603072103 16954184 PMC1564215

[40] Henneke P, Morath S, Uematsu S, Weichert S, Pfitzenmaier M, Takeuchi O, et al (2005) Role of lipoteichoic acid in the phagocyte response to group B *Streptococcus* . J Immunol 174: 6449–6455. 15879147 10.4049/jimmunol.174.10.6449

[41] Bligh EG, Dyer WJ (1959) A rapid method of total lipid extraction and purification. Can J Biochem Physiol 37: 911–917. 13671378 10.1139/o59-099

[42] Walley KR, Lukacs NW, Standiford TJ, Strieter RM, Kunkel SL (1997) Elevated levels of macrophage inflammatory protein 2 in severe murine peritonitis increase neutrophil recruitment and mortality. Infect Immun 65: 3847–3851. 9284162 10.1128/iai.65.9.3847-3851.1997 PMC175549

[43] Wengner AM, Pitchford SC, Furze RC, Rankin SM (2008) The coordinated action of G-CSF and ELR + CXC chemokines in neutrophil mobilization during acute inflammation. Blood 111: 42–49. 10.1182/blood-2007-07-099648 17928531 PMC2575836

[44] Ness TL, Hogaboam CM, Strieter RM, Kunkel SL (2003) Immunomodulatory role of CXCR2 during experimental septic peritonitis. J Immunol 171: 3775–3784. 14500678 10.4049/jimmunol.171.7.3775

[45] Diederich AK, Wobser D, Spiess M Sava IG, Huebner J, Sakinc T (2014) Role of glycolipids in the pathogenesis of *Enterococcus faecalis* urinary tract infection. Plos One 9: e96295 10.1371/journal.pone.0096295 24806450 PMC4012979

[46] Jones CL, Sampson TR, Nakaya HI, Pulendran B, Weiss DS (2012) Repression of bacterial lipoprotein production by *Francisella novicida* facilitates evasion of innate immune recognition. Cell Microbiol 14: 1531–1543. 10.1111/j.1462-5822.2012.01816.x 22632124 PMC3443312

[47] Hilmi D, Parcina M, Stollewerk D, Ostrop J, Josten M, Meilaender A, et al (2014) Heterogeneity of host TLR2 stimulation by *Staphylocoocus aureus* isolates. PLoS ONE 9: e96416 10.1371/journal.pone.0096416 24810614 PMC4014498

[48] Parsons JB, Rock CO (2013) Bacterial lipids: metabolism and membrane homeostasis. Prog Lipid Res 52: 249–276. 10.1016/j.plipres.2013.02.002 23500459 PMC3665635

[49] Jouhet J (2013) Importance of the hexagonal lipid phase in biological membrane organization. Front Plant Sci 4: 494 10.3389/fpls.2013.00494 24348497 PMC3848315

[50] Meiers M, Volz C, Eisel J, Maurer P, Henrich B, Hakenbeck R (2014) Altered lipid composition in *Streptococcus pneumoniae* cpoA mutants. BMC Microbiol 14: 12 10.1186/1471-2180-14-12 24443834 PMC3901891

[51] Lind J, Rämö T, Klement MLR, Bárány-Wallje E, Epand RM, Epand RF, et al (2007) High cationic charge and bilayer interface-binding helices in a regulatory lipid glycosyltransferase. Biochemistry 46: 5664–5677. 10.1021/bi700042x 17444657

[52] Kang JY, Nan X, Jin MS, Youn S-J, Ryu YH, Mah S, et al (2009) Recognition of lipopeptide patterns by Toll-like receptor 2-Toll-like receptor 6 heterodimer. Immunity 31: 873–884. 10.1016/j.immuni.2009.09.018 19931471

[53] Krogh A, Larsson B, von Heijne G, Sonnhammer ELL (2001) Predicting transmembrane protein topology with a hidden Markov model: application to complete genomes. (2001) J Mol Biol. 305: 567–580. 10.1006/jmbi.2000.4315 11152613

[54] Huebner J, Wang Y, Krueger WA, Madoff LC, Martirosian G, Boisot S, et al (1999) Isolation and chemical characterization of a capsular polysaccharide antigen shared by clinical isolates of *Enterococcus faecalis* and vancomycin-resistant *Enterococcus faecium* . Infect Immun 67: 1213–1219. 10024563 10.1128/iai.67.3.1213-1219.1999 PMC96449

[55] Callegan MC, Jett BD, Hancock LE, Gilmore MS (1999) Role of hemolysin BL in the pathogenesis of extraintestinal *Bacillus cereus* infection assessed in an endophthalmitis model. Infect Immun 67: 3357–3366. 10377113 10.1128/iai.67.7.3357-3366.1999 PMC116518

