# Correction: *Enterococcus faecalis* Glycolipids Modulate Lipoprotein-Content of the Bacterial Cell Membrane and Host Immune Response

**DOI:** 10.1371/journal.pone.0136806

**Published:** 2015-08-24

**Authors:** Christian Theilacker, Ann-Kristin Diederich, Andreas Otto, Irina G. Sava, Dominique Wobser, Yinyin Bao, Katrin Hese, Melanie Broszat, Philipp Henneke, Dörte Becher, Johannes Huebner

There is an error in Fig 3. Fig. 2A has been incorrectly repeated. Please view the complete, correct Fig 3 here.

**Fig 3:**
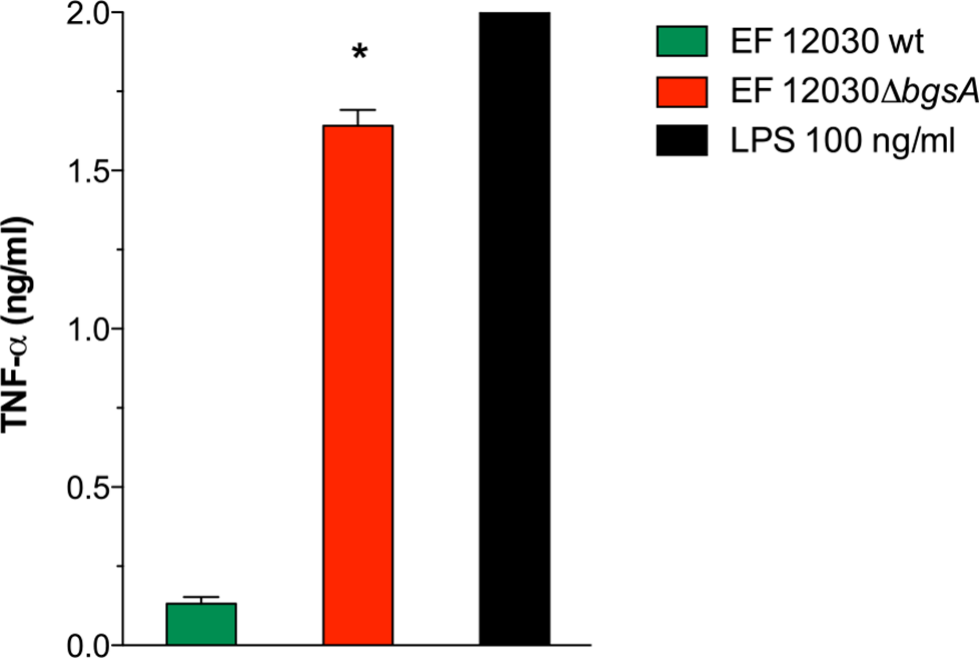
Stimulation of TNF-α production in RAW 264.7 mouse macrophages by lipoprotein-enriched cell membrane fractions of *E*. *faecalis* wild type and *ΔbgsA*. RAW 264.7 cells were incubated with lipoprotein-enriched Triton X-114 extracts from total membrane protein fractions derived from the indicated *E*. *faecalis* strains. The concentration of lipoprotein extracts was measured photometrically and normalized to a bacterial cfu:RAW 264.7 cell ratio of 10,000:1. At 16 h, supernatants were collected and TNF-α concentrations were quantified by ELISA. LPS at a concentration of 100 ng/ml was used as positive control. Data represent mean ± SEM of triplicates. * p < 0.001 12030 *ΔbgsA* versus 12030 WT.
